# Serum hsa-miR-22-3p, hsa-miR-885-5p, Lipase-to-Amylase Ratio, C-Reactive Protein, CA19-9, and Neutrophil-to-Lymphocyte Ratio as Prognostic Factors in Advanced Pancreatic Ductal Adenocarcinoma

**DOI:** 10.3390/cimb47010027

**Published:** 2025-01-03

**Authors:** Jakub Wnuk, Dorota Hudy, Joanna Katarzyna Strzelczyk, Łukasz Michalecki, Kamil Dybek, Iwona Gisterek-Grocholska

**Affiliations:** 1 Department of Oncology and Radiotherapy, Faculty of Medical Sciences in Zabrze, Medical University of Silesia in Katowice, 35 Ceglana St., 40-515 Katowice, Poland; jkb.wnuk@gmail.com (J.W.);; 2 Department of Medical and Molecular Biology, Faculty of Medical Sciences in Zabrze, Medical University of Silesia in Katowice, 19 Jordana St., 41-808 Zabrze, Poland; dorota.hudy@sum.edu.pl (D.H.); jstrzelczyk@sum.edu.pl (J.K.S.); 3 Central Laboratory, University Clinical Center, Medical University of Silesia in Katowice, 14 Medyków St., 40-752 Katowice, Poland

**Keywords:** circulating miRNAs, pancreatic ductal adenocarcinoma, neutrophil–lymphocyte ratio, lipase–amylase ratio, CA19-9, C-reactive protein

## Abstract

Pancreatic cancer (PC) is the seventh most common cause of cancer-related death worldwide. The low survival rate may be due to late diagnosis and asymptomatic early-stage disease. Most patients are diagnosed at an advanced stage of the disease. The search for novel prognostic factors is still needed. Two miRNAs, miR-22-3p and miR-885-5p, which show increased expression in PC, were selected for this study. The aim of this study was to evaluate the utility of these miRNAs in the prognosis of PC. Other prognostic factors such as lipase-to-amylase ratio (LAR), neutrophil-to-lymphocyte ratio (NLR), and carbohydrate antigen 19-9 (CA19-9) were also evaluated in this study. This study was conducted in 50 patients previously diagnosed with pancreatic ductal adenocarcinoma in clinical stage (CS) III and IV. All patients underwent a complete medical history, physical examination, and routine laboratory tests including a complete blood count, C-reactive protein (CRP), CA19-9, lipase, and amylase. Two additional blood samples were taken from each patient to separate plasma and serum. Isolation of miRNA was performed using TRI reagent with cel-miR-39-3p as a spike-in control. Reverse transcription of miRNA was performed using a TaqMan Advanced miRNA cDNA Synthesis Kit. The relative expression levels of miR-22-3p and miR-885-5p were measured using RT-qPCR. Serum hsa-miR-22-3p was detected in 22 cases (44%), while hsa-miR-885-5p was detected in 33 cases (66%). There were no statistically significant differences in serum or plasma miRNA expression levels between patient groups based on clinical stage, gender, or BMI. There were no statistically significant differences in LAR between patients with different CS. For NLR, CRP and CA19-9 thresholds were determined using ROC analysis (6.63, 24.7 mg/L and 4691 U/mL, respectively). Cox’s F test for overall survival showed statistically significant differences between groups (*p* = 0.002 for NLR, *p* = 0.007 for CRP and *p* = 0.007 for CA19-9). Utility as prognostic biomarkers was confirmed in univariate and multivariate analysis for CA19-9, CRP, and NLR. The selected miRNAs and LAR were not confirmed as reliable prognostic markers in PC.

## 1. Introduction

Pancreatic cancer (PC) is the seventh-leading cause of cancer-related death worldwide, causing 466,003 deaths in 2020, with either stable or increasing incidence rates in North America and Europe [1,2]. The 5-year relative survival rate is estimated to be from 6% to 11.5%. The low survival rate might be caused by late diagnosis and the asymptomatic course in the early stages [3,4,5]. The most common type of PC is pancreatic ductal adenocarcinoma (PDAC). It is estimated that it accounts for more than 90% of PC diagnoses [6].

Early diagnosis of PC is of paramount priority for improving treatment outcomes and survival rates. Histopathological analysis is considered the “gold standard” for the diagnosis of PC. Standard diagnostic methods before obtaining histopathology or cytology specimens include endoscopic ultrasonography (EUS), computed tomography (CT), endoscopic retrograde cholangiopancreatography (ERCP), magnetic resonance imaging (MRI), ascites cytology, and exploratory biopsy under laparoscopy or open surgery [7]. As most patients present late, with locally advanced disease or disseminated disease, surgical resection is impossible. Therefore, novel biomarkers related to PC, increasing the probability of early diagnosis and determining the risk of rapid progression, are needed.

Currently, there are several tumor biomarkers: carbohydrate antigen 19-9 (CA19-9), cancer antigen 242 (CA242), carcinoembryonic antigen (CEA), cancer antigen 125 (CA125), microRNAs, and K-RAS gene mutations. Circulating cell-free DNA (cfDNA) and circulating cell-free tumor DNA (CftDNA) are also potential biomarkers [7]. CA19-9 and CEA, diagnostic and prognostic tools in PDAC, with ongoing development and advances in their detection technologies, are still considered basic biomarkers. However, they are not specific to PDAC alone [8]. There are also studies showing that inflammation-specific C-reactive protein (CRP) could be a negative biomarker in PDAC [9]. The serum lipase-to-amylase ratio (LAR), a well-known diagnostic factor in acute pancreatitis that differentiates between alcoholic and non-alcoholic inflammation, has recently been shown to be a useful prognostic factor in resectable pancreatic cancer [10,11].

Other studies have looked at prognostic factors based on immunological cells. These prognostic factors include neutrophil-to-lymphocyte ratio (NLR), platelet-to-lymphocyte ratio (PLR), and lymphocyte-to-monocyte ratio (LMR), with proven utility [12,13].

MiRNAs are short, non-coding, endogenous RNAs (19–24 nucleotides) that are involved in the posttranscriptional regulation of protein synthesis. Therefore, they play a role in the carcinogenesis process and cancer progression as well. Through binding to 3′ untranslated regions (3′ UTRs) of targeted transcripts, the proper miRNAs can cause repression or degradation of a formed transcript through the formation of RNA-induced silencing complexes (RISCs) [14].

It has been proven that circulating microRNAs (miRNAs) might be useful as diagnostic, prognostic, and disease monitoring factors, even more accurate when combined with other PC biomarkers [7,15]. Has-miR-21 is the most widely studied PDAC diagnostic biomarker in terms of microRNAs [16,17,18]. The other commonly evaluated biomarkers are miR-155, miR-196a, and miR-210 [19,20,21,22,23]. In a meta-analysis by Sun et al., the diagnostic accuracy of miRNAs to distinguish PC from healthy controls was assessed. The pooled sensitivity of the included miRNAs was 0.82 (95% CI, 0.81–0.83) and the pooled specificity was 0.76 (95% CI, 0.74–0.78). The analysis included 40 articles with 109 studies. However, this meta-analysis included not only blood-based miRNAs but also tissue, fecal, and pancreatic juice miRNAs, which led to high heterogeneity in the studies. The number of patients with PDAC in the included trials ranged from 11 to 180. Most articles included fewer than 100 PDAC patients (four with the number of PDAC patients exceeding 100). Of these 109 studies, 80 tested the blood-based diagnostic utility of different miRNAs, resulting in a pooled sensitivity of 0.79 (95% CI 0.78–0.80) and a pooled specificity of 0.78 (95% CI 0.76–0.79) [24].

The prognostic value of miRNAs is less commonly evaluated compared to their diagnostic utility. The most commonly tested circulating miRNA is miR-21. Its upregulated expression is associated with poor patient prognosis [16,18,25,26].

Based on our previous review [27], we decided to evaluate the prognostic utility of miR-22-3p and miR-885-5p. MiR-22-3p and miR-885-5p are miRNAs that show increased expression in pancreatic ductal adenocarcinoma, with proven diagnostic utility in several studies [28,29,30]. In a study by Hussein et al., their sensitivity and specificity as diagnostic factors in PDAC were 97.14% and 93.33% for miR-22-3p and 100% and 100% for miR-885-5p, respectively [30].

MiR-22-3p is located on chromosome 17 (17p13.3) and plays roles in various types of cancer, such as lung cancer, cervical cancer, breast cancer, and colon cancer [31,32,33,34]. It is suggested that it targets PLAGL2 or PGC1β to suppress the breast cancer cell tumorigenesis process [35,36]. MiR-885-5p is placed on chromosome 3 (3p25.3). It is suggested that, apart from its role in PDAC, it could play a role in the tumorigenesis of gastric cancer, cholangiocarcinoma, or cervical cancer [37,38,39,40,41].

Therefore, the aim of this study was to determine the prognostic role of several biomarkers—CA19-9, CRP, LAR, NLR, PLR, LMR, miR-22-3p, and miR-885-5p—with the hypothesis that increased levels of the mentioned biomarkers could correlate with poor prognosis in PDAC and possibly create a prognostic model for patients with unresectable pancreatic ductal adenocarcinoma.

## 2. Materials and Methods

### 2.1. Subjects

This study was carried out on 50 patients included in this analysis, who had been previously diagnosed with late PDAC (unresectable—CS III or advanced—CS IV) (pancreatic ductal adenocarcinoma) and referred to the Clinical Oncology Unit of the University Clinical Center of the Medical University of Silesia in Katowice for treatment between April 2022 and April 2024. Patients who had undergone previous pancreatic resection were excluded from the analysis. All patients were subjected to full anamnesis (including past medical history, surgical procedures, and medication intake), physical examination, and routine laboratory testing, including complete blood counts (CBCs), carbohydrate antigen 19-9 (CA-19.9),serum albumin levels, and serum lipase and amylase serum levels, and diagnostic imaging with chest and abdominal CT for complete clinical staging. Based on the CBC results, prognostic factors such as NLR, LMR, and PLR were calculated. LAR was calculated from pre-treatment lipase and amylase levels. Data concerning patients’ body mass index (BMI)and performance status evaluated using their ECOG score (Eastern Cooperative Oncology Group score) were included as well. Overall survival (OS) was calculated as the length of time from the date of PDAC diagnosis to the date that the patients were still alive or the patient’s death. The demographic data are presented in Table 1.

This study was approved by the Local Ethics Committee of the Medical University of Silesia in Katowice by decree no. PCN/CBN/0022/KB1/232/32 of 16 November 2021. Informed consent was obtained from all participants.

### 2.2. Blood Specimen Collection and Handling for miRNA Isolation

Two 5-milliliter blood samples were taken from each patient by a single person. Blood samples were taken within 1–7 days before the start of treatment, between 7 and 8 a.m. One sample was collected in a tube with sodium citrate to separate the plasma. The second was collected in a tube without an anticoagulant and allowed to stand for 30–45 min before being centrifuged to separate the serum. The samples were handled by two selected and trained personnel. Both samples were centrifuged at 2000× *g* for 10 min at 4 °C within 1–2 h of collection and stored at −80 °C until further use.

### 2.3. Isolation of miRNA

Serum and plasma before isolation were kept at −80 °C. The isolation of miRNA was carried out with TRI Reagent (Sigma-Aldrich, St. Louis, MI, USA, nr cat.: T3809). Approximately 750 µL of TRI was mixed with 250 µL of serum and plasma, and 1.5 µL of cel-miR-39-3p spike-in (33 fmol/µL) was added (NorgenBiotek Corp., Thorold, ON, Canada, nr cat.: #59000). This mixture was mixed by inverting the probe upside down several times and transferred onto 5PRIME heavy phase lock gel (Quantabio, Beverly, MA, USA, nr cat.:2302830) followed by adding 0.1 mL of 1-bromo-3-chloropropane (Sigma-Aldrich, St. Louis, MI, USA, nr cat.: B9673). The mixture was vigorously shaken, incubated for 5 min at room temperature, and then centrifuged for 15 min at 12,000× *g* at 4 °C. The aqueous phase was transferred to a new Eppendorf tube and 0.5 mL of isopropanol was added and incubated for 10 min at RT and then centrifuged for 8 min at 12,000× *g* at 4 °C. The supernatant was removed and the pellet was washed with 75% ethanol, vortexed, and centrifuged at 12,000× *g* at 4 °C. Then, the pellet was air-dried for about 10 min at RT, dissolved with 15 µL of RNase-free water, and stored at −80°C.

### 2.4. Reverse Transcription of miRNA

Reverse transcription of miRNA was performed with a TaqMan Advanced miRNA cDNA Synthesis Kit (Thermo Fisher Scientific, Waltham, MA, USA, nr cat.: A28007) using 2 µL of isolated miRNA on a Mastercycler personal instrument (Eppendorf, Hamburg, Germany). The composition of the reaction mixtures and reaction conditions are shown in Supplementary Table S1.

### 2.5. Real-Time PCR

The relative expression of miRNA in serum/plasma was determined with TaqMan microRNA assays for hsa-miR-22-3p (Assay ID: 477985_mir), hsa-miR-885-5p (Assay ID: 478207_mir), and cel-miR-39-3p (Assay ID: 478293_mir). RT-qPCR was performed on a Quant Studio 5 instrument (Thermo Fisher Scientific, Waltham, MA, USA) using TaqMan Fast Advanced Master Mix (Thermo Fisher Scientific, Waltham, MA, USA, nr cat.: 4444964). The reaction mixture consisted of a 10 µL master mix, 1 µL of primers, 4 µL of water, and 5µLof microRNA cDNA which was diluted 5 times. The reaction was performed in triplicate and the reaction conditions were as follows: 95 °C for 20 s, 40 × 1 s at 95 °C, and 20 s at 60 °C. Relative expression was calculated with the ΔΔCT method where cel-miR-39-3p was treated as a reference and calibrated with the non-isolated spike-in control.

### 2.6. Statistical Analysis

Data were analyzed using Statistica 13.3 Software (TIBCO Software Inc, Palo Alto, CA, USA). *p*-values < 0.05 were accepted as statistically significant. The chi-square test and its variants were used for qualitative variables to compare the different groups. The Mann–Whitney U test was used to compare quantitative variables with a non-parametric distribution, while Student’s *t*-test was used to compare quantitative variables with a parametric distribution. To compare multiple independent groups of quantitative variables with non-parametric distributions, the Kruskall–Wallis ANOVA test was used. The correlations between quantitative variables were analyzed using Spearman’s rank correlation coefficient.

Thresholds were estimated using receiver operating characteristic curve analysis, distinguishing patients who survived longer than the median overall survival (OS), which was 258 days. The likelihood of survival was analyzed using the Kaplan–Meier estimator and Cox’s F test.

Figures showing Kaplan–Meier survival probability curves were calculated using R 4.4.2: A language and environment for statistical computing (R Foundation for Statistical Computing, Vienna, Austria).

## 3. Results

**Figure 1:**
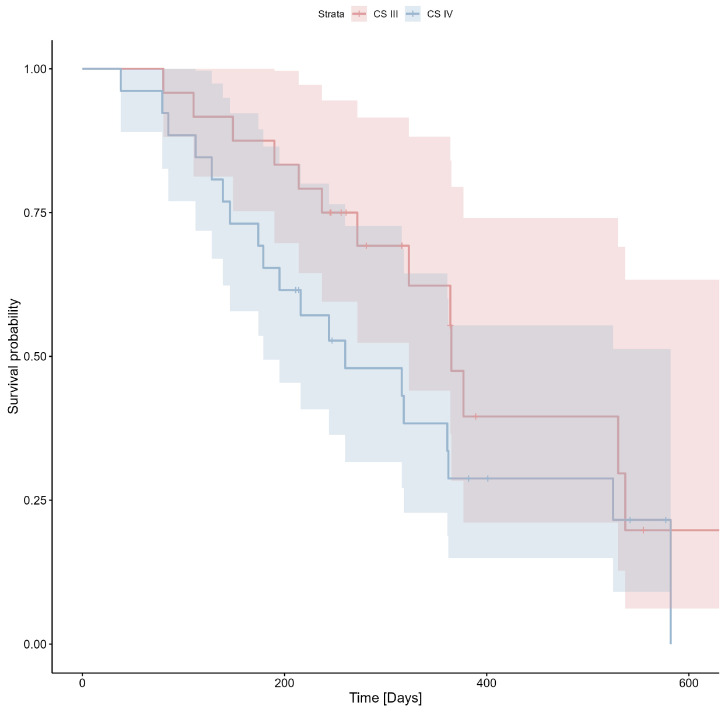
Probability of survival according to clinical stage (Cox’s F test *p* = 0.043). CS—clinical stage.

The median overall survival among the 50 patients included was 258 days (IQR: 190–365 days), with 32 patients (64%) completely followed. The likelihood of survival in the clinical stage III and IV groups is shown in Figure 1.

### 3.1. Age, BMI, and Sex

The median age of the included patients was 67.5 years with an IQR of 60–72 years. Age was correlated with observed overall survival (Spearman’s rho-coefficient −0.4 with *p* = 0.004). Using ROC analysis, a threshold age of 72 years was identified as the cut-off point for poor prognosis (Youden index 0.38).

**Figure 2:**
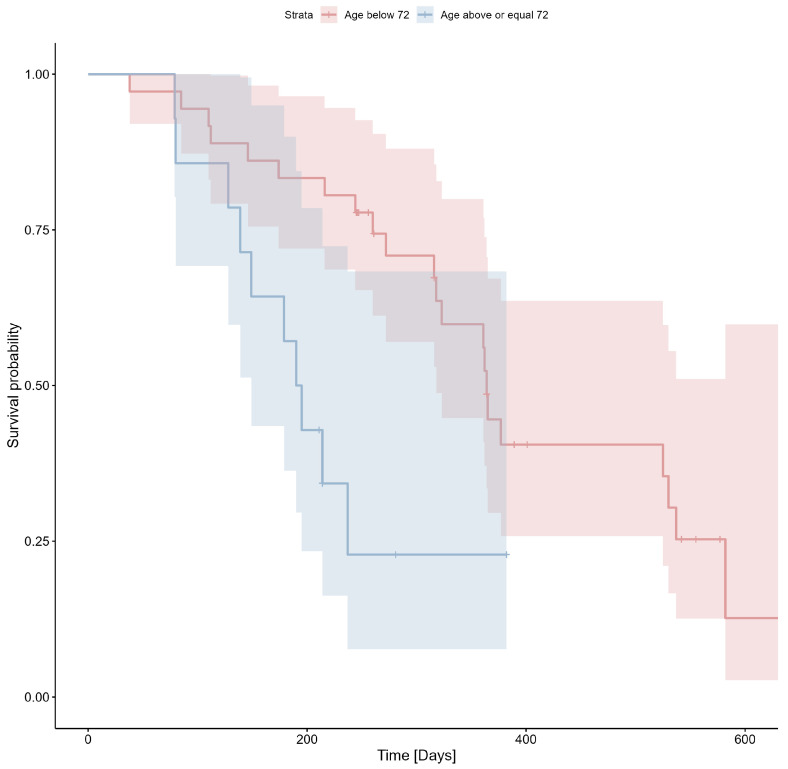
Probability of survival based on an age threshold of 72 years (Cox’s F test *p* > 0.05; 0.07).

The likelihood of survival according to age is shown in Figure 2.

There was no statistically significant correlation between BMI and overall survival (Spearman’s rho, with *p* > 0.05).

There were no statistically significant differences in survival between male and female patients, with Cox’s F test *p* > 0.05.

### 3.2. CA19-9

Carbohydrate antigen 19-9 was evaluated in 50 patients with a median value of 782.2 U/mL (IQR: 46.73–3236 U/mL). There were statistically significant differences between patients with different clinical stages (*n* = 25, median 369 U/mL, IQR 41.46–1028.45 U/mL and *n* = 25, median 1596 U/mL, IQR 68.3–6098 U/mL for CS III and CS IV, respectively; Mann–Whitney U test *p* = 0.043). There were also no statistically significant correlations between CA19-9 levels and variables such as BMI and age (Spearman’s rho with *p* > 0.05).

**Figure 3:**
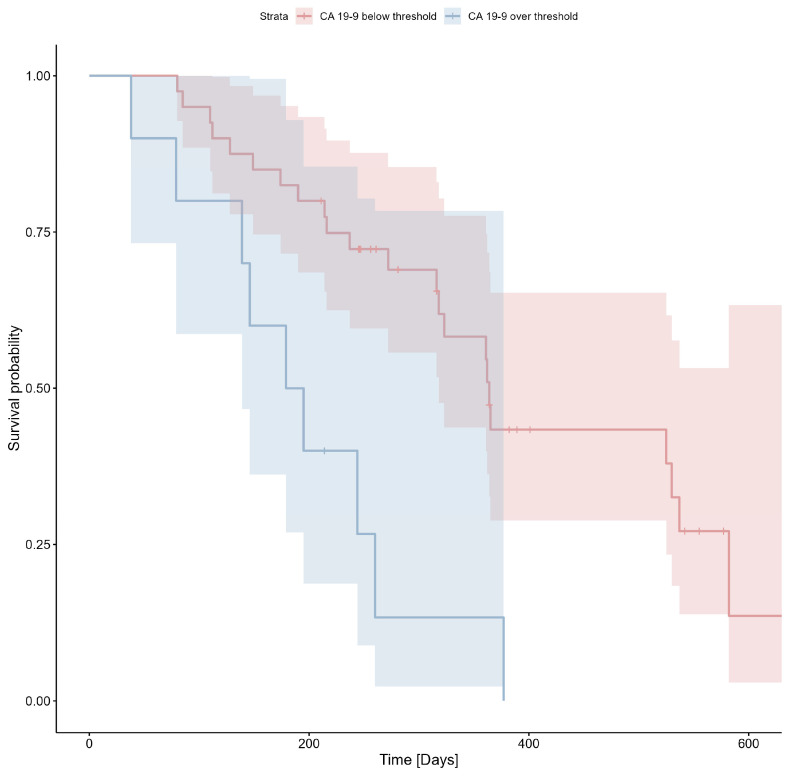
Probability of survival based on CA19-9 levels (threshold: 4619 U/mL).

The cut-off value of 4691 U/mL was determined using ROC analysis (Youden index = 0.34) and applied to the survival analysis. The Kaplan–Meier estimator for CA19-9 is shown in Figure 3; Cox’s F test *p* = 0.007.

Sex, performance status, tumor size, and nodal status on the TNM scale did not have a statistically significant effect on the levels of CA19-9 tested (Supplementary Table S2).

### 3.3. CRP

**Figure 4:**
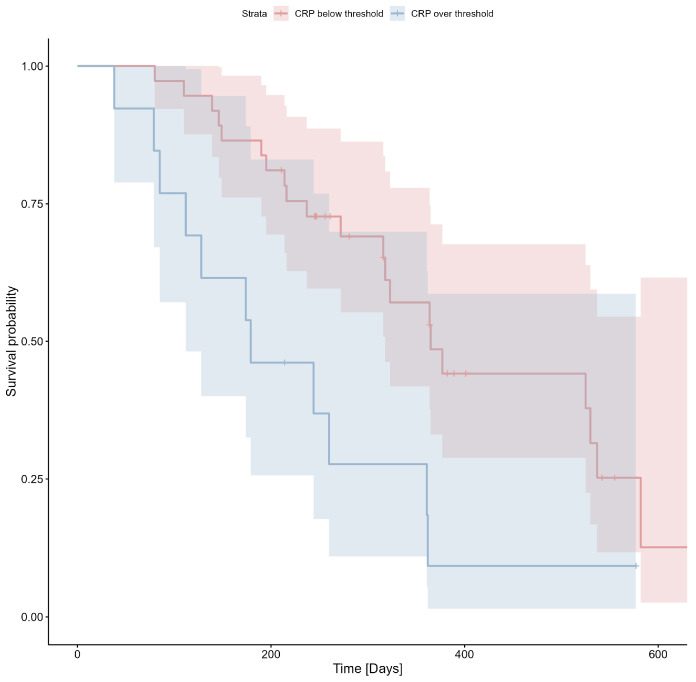
Probability of survival based on C-reactive protein (CRP) level (threshold 24.7 mg/L).

C-reactive protein levels were assessed in 48 patients with a median value of 5.65 mg/dL (IQR: 2.65–29.7 mg/dL). There were statistically significant differences between patients with different clinical stages (*n* = 23, median 2.9 mg/dL, IQR 1.8–14.6 mg/dL and *n* = 25, median 27.8 mg/dL, IQR 4.4–60.8 mg/dL for CS III and CS IV, respectively; Mann–Whitney U test *p* = 0.002). There were no statistically significant correlations between CRP levels and variables such as BMI and age (Spearman’s rho with *p* > 0.05). There were statistically significant differences in CRP levels between patients with a history of pulmonary disease (asthma or chronic obstructive pulmonary disease) and the rest of the patients. Median CRP levels were 37.8 mg/dL (*n* = 4; IQR: 28.8–113.3 mg/dL) in patients with a history of pulmonary disease and 5.5 mg/dL (*n* = 44; IQR: 2.6–24.2 mg/dL) in patients without a history of pulmonary disease (Mann–Whitney U test *p* = 0.03). Detailed data are presented in Supplementary Table S2. The cut-off value of 24.7 mg/L was determined using ROC analysis (Youden index = 0.29) and applied to the survival analysis. The Kaplan-Meier estimator for CRP is shown in Figure 4; Cox’s F test *p* = 0.007.

Patients with worse performance status tended to have higher CRP levels with *p* = 0.031 in the Kruskal–Wallis ANOVA test. Patients with higher T status in TNM score tended to have lower CRP levels with *p* = 0.02 in the Kruskal–Wallis ANOVA test (Supplementary Table S2).

### 3.4. Albumin

Albumin levels were available for 45 patients with a mean value of 3.97 g/dL (min.–max. range: 2.66–4.97 g/dL).There were no statistically significant differences between patients with different clinical stages (*n* = 21, mean 4.05 g/dL, min.–max. range 2.96–4.87 g/dL and *n* = 24, mean 3.9 g/dL, min.–max. range 2.66–4.97 g/dL for CS III and CS IV, respectively; Student’s *t*-test *p* > 0.05). There were also no statistically significant correlations between albumin levels and variables such as BMI and age (Spearman’s rho with *p* > 0.05).

### 3.5. Serum Lipase and Amylase, LAR

Serum lipase and amylase levels were available for 49 patients. The values of these biomarkers by clinical stage are shown in Table 2.

Lipase-to-amylase ratio was calculated based on the reported results with a median value of 0.69 (IQR: 0.48–1.12). There were statistically significant differences between patients with different clinical stages (*n* = 23, median 0.63, IQR 0.43–1.28 and *n* = 26, median 0.73, IQR 0.48–1.12 for CS III and CS IV, respectively; Mann–Whitney U test *p* > 0.05). There were also no statistically significant correlations between LAR levels and variables such as BMI and age (Spearman’s rho with *p* > 0.05).Median LAR values were statistically significantly higher in patients with a history of diabetes (*n* = 19; 0.98; IQR: 0.53–1.35) compared to median LAR values in patients without diabetes (*n* = 30; 0.60; IQR: 0.43–0.76) with the Mann–Whitney U test *p* = 0.03. Detailed information is shown in Supplementary Table S2.

### 3.6. NLR, PLR, and LMR

A complete blood count was performed in all 50 patients. The results of the parameters based on the CBC are shown in Table 3.

**Figure 5:**
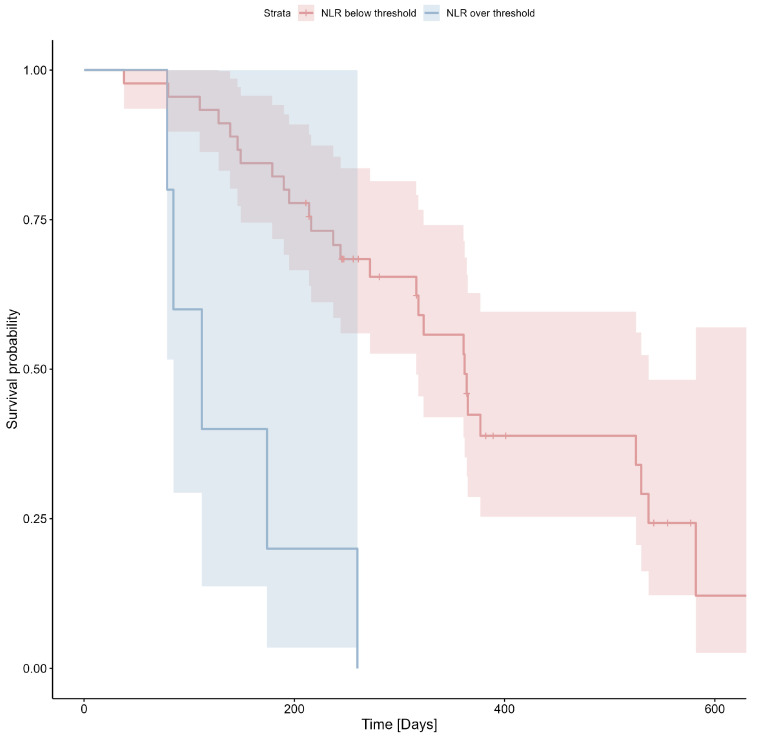
Probability of survival based on NLR values (threshold: 6.63).

The threshold of 6.63 for NLR was determined using ROC analysis (Youden index = 0.23) and applied to the survival analysis. The Kaplan–Meier estimator for NLR is shown in Figure 5; Cox’s F test *p* = 0.002.

### 3.7. hsa-miR-22-3p

Serum hsa-miR-22-3p was detected in 22 cases (44%) with 2 cases of overestimated results due to late expression during control microRNA PCR, while plasma hsa-miR-22-3p was detected in 20 cases (40%) with 4 cases of overestimated results due to late expression during control microRNA PCR. Overestimated cases were excluded from further analysis. The differences in serum/plasma expression were not statistically significant (chi-squared test *p* > 0.05).

The median expression levels of serum and plasma hsa-miR-22-3p depending on the factors tested are shown in Table 4.

There were no statistically significant differences between expression levels based on sample source (Mann–Whitney U test *p* > 0.05).

The expression of has-miR-22-3-p was not statistically significantly correlated with BMI and age, regardless of sample source (Spearman’s rho with *p* > 0.05).

Median serum hsa-miR-22-3p expression levels were statistically significantly higher in patients on ongoing anticoagulant therapy (*n* = 4; 2.717; IQR: 1.39–4.23) compared to the remaining patients (*n* = 16; 0.269; IQR: 0.021–0.743), Mann–Whitney U test *p* = 0.01. There were no such observations for plasma has-miR-22-3p expression levels. Detailed information is shown in Supplementary Table S2.

### 3.8. hsa-miR-885-5p

For hsa-miR-885-5p, it was detected in serum in 33 cases (66%) with 4 cases of overestimated results due to late expression during PCR of control miRNA and in plasma in 28 cases (56%) with 1 case of overestimated results due to late expression during PCR of control miRNA. The differences in serum/plasma expression were not statistically significant (chi-square test *p* > 0.05).

Table 4 shows the median expression levels of hsa-miR-885-5p in serum and plasma depending on the factors tested.

### 3.9. Multivariate Analysis

The Cox proportional hazards model was used to investigate the association between the overall survival of patients and selected predictor variables (age, clinical stage, CA19-9 levels, CRP levels, and NLR) as a multivariate analysis. The chi-squared *p*-value for the obtained model was 0.0004, indicating that the variables used in the analysis were associated with overall survival. CA19-9, CRP, and NLR were independent prognostic factors in PDAC. Based on beta coefficient values, NLR was the best prognostic factor among them. The detailed results of the prognostic model are shown in Table 5.

The assumptions of the test were tested using proportional hazard assumption (PH) and scaled Schoenfeld residuals. The results are presented in Supplementary Table S3 and Supplementary Figures S1–S5.

## 4. Discussion

The majority of patients with PDAC are diagnosed with locally advanced (unresectable) or advanced (metastatic) disease. Median overall survival in historical data ranged from 3 to 92 weeks and has been extended by the use of novel systemic therapies, starting with gemcytabine (median OS 6.8 months), gemcytabine + nab-paclitaxel (median OS 8.5 months), and FOLFIRINOX (median OS 11.1 months) regimens [42,43,44]. The search for prognostic factors in advanced PDAC is still needed.

The cut-off values for various biomarkers used in this study vary from literature reports. NLR cut-off values can vary from 2 to 5 [45], PLR cut-off values can vary from 100 to 225 [46],and LMR cut-off values can vary from 2.05 to 4.62 [47]. CRP thresholds in other studies have fluctuated around values of 3–4.5 mg/dL, but these studies were conducted in patient cohorts that included resectable PDAC [48,49]. For CA19-9, the threshold in our study was higher than in other studies, but this may be due to the smaller number of patients enrolled in this study [50].

CA19-9 has been a well-established prognostic factor in unresectable PDAC since the 2005 study by Maisey et al. In their study, patients with CA19-9 levels above the median value were examined using ROC curve analysis based on median OS. Such results were later confirmed by several other studies [51,52,53]. Modern studies are combining CA19-9 with other biomarkers, such as NLR or miRNA, in an attempt to improve the prognostic value of such biomarkers [54,55,56].

C-reactive protein is an acute-phase protein produced in the liver after tissue damage, infection, or other inflammatory stimuli. This study provides evidence that CRP may be a prognostic factor in unresectable PDAC that warrants further investigation. This is in line with the recent study by Bonazzi et al., who investigated CRP expression in PDAC tissue [9]. Similar results have been seen in previous studies in both operable and inoperable PDAC [48,49]. Higher CRP levels were associated with poorer patient performance status. This observation is in line with other studies. Inflammation is considered a factor that reduces appetite and is associated with poorer physical function [57,58]. High levels of C-reactive protein were also more common in patients with T2 tumors according to the TNM score. However, four out of six of these patients presented with metastases, and CRP levels were also associated with a higher clinical stage, so it is more likely that high CRP levels are associated with a more advanced clinical stage of the disease. However, as an acute-phase protein, CRP levels can be influenced by various inflammatory conditions. In this study, lung diseases (asthma and chronic obstructive pulmonary disease) were associated with higher CRP levels. This should be taken into account before using CRP as a prognostic biomarker.

Malnutrition and cachexia are common diagnoses in oncology. In advanced PDAC, malnutrition can progress during treatment [59]. General epidemiological studies and recent studies in PDAC have shown that low albumin levels are a negative prognostic factor associated with malnutrition [60,61]. This study did not show similar results, probably due to the rather homogeneous results of albumin levels among the patients enrolled.

The lipase-to-amylase ratio (LAR) is used to differentiate between alcoholic and non-alcoholic acute pancreatitis. A recent meta-analysis reported that an LAR greater than 3 has moderate accuracy in diagnosing alcoholic pancreatitis [10]. In the study by Stotz et al., LAR was used as a prognostic factor in PDAC. It was shown that a preoperative LAR above 2.74 was a poor prognostic factor. The same study did not provide similar evidence for metastatic PDAC [11]. In our study, we evaluated the benefit of LAR in the setting of unresectable PDAC (CS III and IV). The results are in line with the Stotz study. In addition, this study provides data that LAR levels may be influenced by diabetes. Patients with diabetes tended to have higher LAR levels than the other patients. This is consistent with other studies. According to the study by Hameed et al., lipase levels can be increased by many diseases, including diabetes [62]. Interestingly, these differences were not observed between patients treated for diabetes. According to the study by Cakmak et al., there were no differences in amylase or lipase levels in patients on incretin-based therapies. The only differences were observed in patients using insulin secretagogues, which cause higher amylase and lipase levels [63].

The prognostic role of NLR, PLR, or LMR has been determined in a few studies in recent years [64,65,66,67,68]. Tumor growth is the cause of local inflammation, which leads to the escalation of tumor progression. Inflammation is related to the chemotaxis of various immunologic cells such as neutrophils, macrophages, lymphocytes, and mastocytes, which through the expression of cytokines affect local immunologic response and tumor growth [69,70].This study provides evidence that NLR may be a strong and independent prognostic factor in PDAC. However, compared with other studies, the cut-off value of NLR is still unclear and varies from study to study.

MiRNAs play important roles in the carcinogenesis and development of PDAC. The minimally invasive and painless sampling procedure, with its low risk of complications, makes miRNAs potentially good candidates for biomarkers. Circulating miRNAs are considered stable molecules; however, proper measures should be taken during specimen sampling and handling.

Proper sampling and experimental protocols are important for the validity of the results. MiRNAs’ expression is naturally diverse, which may affect the results of any planned study [71]. The choice of blood sample type may be fundamental to the study results. In a study by Wang et al., miRNA expression was higher in serum samples compared to corresponding plasma samples. This phenomenon was explained by the coagulation process, which may increase sample-to-sample variation. According to this study, plasma is the sample of choice for studying circulating miRNA expression [72].

In this study, miRNA levels were not associated with patient age, gender, or BMI. However, there were noticeable trends in relative hsa-miR-22-3p and hsa-miR-885-5p expression levels based on patient gender. This is in contrast with the study by Ameling et al., which showed a strong association between miRNA expression levels and the above factors [73].

Regarding medication use, we found that patients receiving anticoagulant therapy tended to have a higher relative expression of serum hsa-miR-22-3p compared to patients not receiving anticoagulant therapy. Drug intake is considered as a factor that may interfere with miRNA expression. In the study by de Boer et al., aspirin use led to reduced levels of circulating miR-126, as platelets are considered to be a major source of circulating miR-126 [74]. The differences in serum hsa-miR-22-3p relative expression between groups with and without anticoagulation require further study in larger patient populations, especially since this phenomenon was not observed in the plasma hsa-miR-22-3p group.

All of the abovementioned inconsistencies could be explained by the small number of patients and relatively low expression levels of selected miRNAs in this study. In the study by Witwer and Halushka, the variability between studies on miRNAs was frequently mentioned and large patient groups were recommended to guarantee statistical significance [75].

Disease CS and tumor size are also factors mentioned in many studies that may be associated with miRNA expression in patients with PDAC, either with upregulated [16,20,76,77,78,79,80,81,82] and downregulated [83,84,85] miRNAs. Therefore, in most of these studies, miRNA expression level is considered as an effective and efficient biomarker for early PC diagnosis.

The prognostic value of different circulating miRNAs in PDAC seems well established. The meta-analysis by Guraya S. et al. provides data that miR-21 is a useful prognostic biomarker in digestive system neoplasms. In PDAC, a pooled HR for worse OS was observed (3.77, 95% CI 1.63-8.73, *p*-value < 0.01). However, the high heterogeneity of the included studies was a limitation of this analysis. In addition, the studies included in this analysis were mostly based on tissue samples. The number of studies included ranged from 65 to 229 [86]. In our previous review, we found 21 studies on circulating miRNAs in PDAC that were useful as prognostic factors. The most commonly tested miRNAs were hsa-miR-21 and hsa-miR-196a. The studies were based on PDAC patient populations ranging from 20 to 197, with 14 studies including more than 100 PDAC patients, where miRNAs were tested in blood samples [27]. In the review by Wald et al., miRNAs in PDAC had prognostic value, especially in tissue-based samples. As in the previous studies, hsa-miR-21 was the most frequently tested biomarker. However, only a few miRNAs have been identified as prognostic factors due to differences in patient populations, study designs, and methodologies [87].

In this study, we tried to evaluate the utility of hsa-miR-22-3p and hsa-miR-885-5p as prognostic factors in unresectable pancreatic cancer (CS III and CS IV). The studies that provided information on their utility as diagnostic factors were based on small cohorts of patients (11 and 35 patients) with resectable PDAC (CS IIA-IIB). In addition, the control mRNAs used in these studies and in our study were different (a mixture of 10 synthetic miRNAs, endogenous miR-3196, and exogenous cel-miR-39-3p) [29,30]. Therefore, it is difficult to compare the results of these studies. The relative expression levels of the selected mRNAs were low in our study, so the analysis of their usefulness as a prognostic factor was not reliable.

There are several barriers to the use of miRNAs as clinically reliable biomarkers. Preclinical bias may be caused by improper sample handling, leading to cell lysis and contamination by cellular miRNA fractions. BMI, gender, and age could also influence the results of the tests [73,88]. Other difficulties relate to the methods used as expression control assays. The spike-in control is an exogenous small RNA molecule added to the sample before extraction, usually from an unrelated species such as *Caenorhabditis elegans*, whereas endogenous controls are based on native miRNAs, which are considered stable and not affected by the patient’s cellular miRNA state. The study design determines the choice between these methods and no single standard is used [89,90]. Therefore, in our opinion, the clinical application of miRNAs as prognostic tools should be approached with caution, as the miRNAs selected in this study have no utility in clinical practice. However, other miRNAs such as miR-21, miR-196a, and miR-373 have been frequently evaluated for their prognostic utility in PDAC. Therefore, more research is needed.

## 5. Conclusions

CA19-9, CRP, NLR, and patient age were found to be independent prognostic factors in unresectable PDAC in this study. LAR is not a potential prognostic biomarker in unresectable PDAC. This study could not provide data on hsa-miR-22-3p and hsa-miR-885-5p levels as prognostic factors in PDAC. The small number of patients in the study may have influenced the results and reduced the reliability of the derived results. We recommend that miRNA studies in relatively rare neoplasms be planned using a multicenter approach and with longer enrolment periods in order to obtain larger patient cohorts to increase the reliability of the planned studies. Standardization of extraction techniques, sample handling, and study methodology could increase the reliability of miRNA profiling in clinical practice. Further studies are encouraged to provide novel prognostic biomarkers, with larger patient groups enrolled in the studies.

### Limitations

The limitations of this study are mostly connected to the low number of participants recruited.

## Figures and Tables

**Table 1:** Demographic characteristics of included patients. BMI (body mass index), ECOG (Eastern Cooperative Oncology Group score), CS (clinical stage), IQR—interquartile range, FOLFIRINOX—5-fluorouracil + irinotecan + oxaliplatin, GCB—gemcytabine, and nab-PXL + GCB—nab-paclitaxel + gemcytabine.

Patient Characteristics	General	Male	Female	*p*-Value
Number of participants	50	22 (44%)	28 (56%)	
Age [years]	Median: 67.5 [years] IQR60–72.0	Median: 67.5 [years] IQR 63–70	Median: 68 [years] IQR60–72.5	Mann–Whitney U test *p* > 0.05
BMI [kg/m^2^]	Median: 23.19 [kg/m^2^] IQR 21.2–25	Median: 23.74 [kg/m^2^] IQR 21.46–24.85	Median: 21.91 [kg/m^2^] IQR 18.99–26.06	Mann–Whitney U test *p* > 0.05
T stage *n* (% of patient population)	T2 = 6 (12%) T3 = 10 (20%) T4 = 34 (68%)	T2 = 2 (9.09%) T3 = 5 (22.73%) T4 = 15 (68.18%)	T2 = 4 (14.29%) T3 = 5 (17.86%) T4 = 19(67.85%)	Mann–Whitney U test *p* > 0.05
N stage *n* (% of patient population)	N0 = 19 (38%) N1 = 21 (42%) N2 = 6 (12%) N3 = 2 (4%) lack of data = 2 (4%)	N0 = 10 (45.45%) N1 = 10 (45.45%) N2 = 1 (4.54%) N3 = 1 (4.54%)	N0 = 9 (32.14%) N1 = 11 (39.29%) N2 = 5 (17.86) N3 = 1 (3.57%) lack of data = 2 (7.14%)	Mann–Whitney U test *p* > 0.05
M stage *n* (% of patients population)	M0 = 24 (48%) M1 = 26 (52%)	M0 = 9 (40.91%) M1 = 13 (59.09%)	M0 = 15 (53.57%) M1 = 13 (46.43%)	Yate’s test *p* > 0.05
CS *n* (% of patiennt population)	III = 24 (48%) IV = 26(52%)	III = 9 (40.91%) IV = 13 (59.09%)	III = 15 (53.57%) IV = 13 (46.43%)	Yate’s test *p* > 0.05
ECOG *n* (% of patient population)	0 = 11 (22%) 1 = 26 (52%) 2 = 10 (20%) 3 = 3 (6%)	0 = 5(22.73%) 1 = 13 (59.09%) 2 = 3 (13.64%) 3 = 1 (4.54%)	0 = 6 (21.43%) 1 = 13 (46.43%) 2 = 7 (25%) 3 = 2 (7.14%)	Mann–Whitney U test *p* > 0.05
History of cholecystectomy	Yes [*n* = 9] (18%) No [*n* = 41] (82%)	Yes [*n* = 3] (13.64%) No [*n* = 19] (86.36%)	Yes [*n* = 6] (21.43%) No [*n* = 22] (78.57%)	Yate’s test *p* > 0.05
History of palliative pancreatic bypass surgery	Yes [*n* = 4] (8%) No [*n* = 46] (92%)	Yes [*n* = 1] (4.55%) No [*n* = 21] (95.46%)	Yes [*n* = 3] (10.71%) No [*n* = 25] (89.29%)	Yate’s test *p* > 0.05
History of biliary stent placement	Yes [*n* = 24] (48%) No [*n* = 26] (52%)	Yes [*n* = 7] (31.82%) No [*n* = 15] 68.18%)	Yes [*n* = 17] (39.29%) No [*n* = 11] (60.71%)	Yate’s test *p* > 0.05 (0.08)
History of pulmonary disease	Yes [*n* = 4] (8%) No [*n* = 46] (92%)	Yes [*n* = 2] (9.09%) No [*n* = 20] (90.91%)	Yes [*n* = 2] (7.14%) No [*n* = 46] (92.86%)	Yate’s test *p* > 0.05
History of diabetes	Yes [*n* = 19] (38%) No [*n* = 31] (62%)	Yes [*n* = 10] (45.45%) No [*n* = 12] (54.55%)	Yes [*n* = 9] (32.14%) No [*n* = 19] (67.86%)	Yate’s test *p* > 0.05
History of cardiovascular disease	Yes [*n* = 37] (74%) No [*n* = 13] (26%)	Yes [*n* = 15] (68.18%) No [*n* = 7] (31.82%)	Yes [*n* = 22] (78.57%) No [*n* = 6] (21.43%)	Yate’s test *p* > 0.05
History of anticoagulant medication intake	Yes [*n* = 10] (20%) No [*n* = 40] (80%)	Yes [*n* = 8] (36.36%) No [*n* = 14] (63.64%)	Yes [*n* = 2] (7.14%) No [*n* = 26] (92.86%)	Yate’s test *p* = 0.027
History of hypotensive medications	Yes [*n* = 32] (64%) No [*n* = 18] (36%)	Yes [*n* = 11] (50%) No [*n* = 11] (50%)	Yes [*n* = 21] (25%) No [*n* = 7] (75%)	Yate’s test *p* > 0.05 (0.08)
History of oral hypoglycemic medications	Yes [*n* = 13] (26%) No [*n* = 37] (74%)	Yes [*n* = 8] (36.36%) No [*n* = 14] (63.64%)	Yes [*n* = 5] (17.86%) No [*n* = 23] (82.14%)	Yate’s test *p* > 0.05
History of alcohol usage	Yes [*n* = 9] (18%) No [*n* = 41] (82%)	Yes [*n* = 4] (18.18%) No [*n* = 18] (81.82%)	Yes [*n* = 5] (17.86%) No [*n* = 23] (82.14%)	Yate’s test *p* > 0.05
History of smoking	Yes [*n* = 21] (42%) No [*n* = 29] (58%)	Yes [*n* = 9] (40.91%) No [*n* = 13] (59.09%)	Yes [*n* = 12] (42.86%) No [*n* = 16] (57.14%)	Yate’s test *p* > 0.05
Systemic therapy administered	FOLFIRINOX = 29 (58%) GCB = 11 (22%) nab-PXL + GCB = 6 (12%) haven’t started = 4 (8%)	FOLFIRINOX = 14 (63.64%) GCB = 4 (18.18%) nab-PXL + GCB = 3 (13.64%) haven’t started = 1 (4.54%)	FOLFIRINOX = 15 (53.56%) GCB = 7 (25%) nab-PXL + GCB = 3 (10.72%) haven’t started = 3 (10.72%)	

**Table 2:** Differences in serum lipase and amylase levels. CS—clinical stage.

Biomarker	Biomarker Serum Levels (U/L) *n* = 49	Biomarker Serum Levels CS III (U/L) *n* = 23	Biomarker Serum Levels CS IV (U/L) *n* = 26	*p*-Value
Lipase	Median: 32.6 IQR: 18.6–48.7	Median: 36.8 IQR: 19.6–54.6	Median: 26.9 IQR: 15.0–44.3	Mann–Whitney U test *p* > 0.05
Amylase	Median: 41.9 IQR: 31.5–75.5	Median: 44.9 IQR: 35.7–73.8	Median: 41.4 IQR: 26.5–76.2	Mann–Whitney U test *p* > 0.05

**Table 3:** Neutrophil-to-lymphocyte ratio (NLR), platelet-to-lymphocyte ratio (PLR), and lymphocyte-to-monocyte ratio (LMR) based on clinical stage.

Parameter	General *n* = 50	CS III *n* = 24	CS IV *n* = 26	*p*-Value
Neutrophil-to-lymphocyte ratio (NLR)	Median: 2.71 IQR: 2.12–4.25	Median: 2.46 IQR: 2.05–3.15	Median: 3.69 IQR: 2.26–5.83	Mann–Whitney U test *p* = 0.043
Platelet-to-lymphocyte ratio (PLR)	Median: 132.66 IQR: 97.26–171.55	Median: 129.85 IQR: 97.26–161.68	Median: 155.68 IQR: 110.63–233.82	Mann–Whitney U test *p* > 0.05
Lymphocyte-to-monocyte ratio (LMR)	Median: 2.94 IQR: 1.81–3.81	Median: 3.42 IQR: 2.11–3.85	Median: 2.36 IQR: 1.29–3.59	Mann–Whitney U test *p* > 0.05

There were also no statistically significant correlations between NLR, PLR, and LMR values and BMI or age (Spearman’s rho with *p* > 0.05).

**Table 4:** Results of hsa-miR-885-5p expression and hsa-miR-22-3p expression.

Sample	General Expression Levels	Sex		Clinical Stage	
Serum hsa-miR-885 (*n* = 29)	Median: 0.030 IQR: 0.009–0.523	Male (*n* = 14)	Female (*n* = 15)	CS III (*n* = 14)	CS IV (*n* = 15)
		Median: 0.013 IQR: 0.0003–0.045	Median: 0.05 IQR: 0.011–0.735	Median: 0.013 IQR: 0.0003–0.523	Median: 0.045 IQR: 0.012–0.735
		Mann–Whitney U test *p* > 0.05		Mann–Whitney U test *p* > 0.05	
Plasma hsa-miR-885 (*n* = 27)	Median: 0.082 IQR: 0.002–0.305	Male (*n* = 12)	Female (*n* = 15)	CS III (*n* = 15)	CS IV (*n* = 12)
		Median: 0.037 IQR: 0.003–0.261	Median: 0.051 IQR: 0.011–0.735	Median: 0.019 IQR: 0.0007–0.142	Median: 0.256 IQR: 0.03–1.271
		Mann–Whitney U test *p* > 0.05		Mann–Whitney U test *p* > 0.05 (0.053)	
Serum hsa-miR-22-3p (*n* = 20)	Median: 0.373 IQR: 0.087–1.05	Male (*n* = 7)	Female (*n* = 13)	CS III (*n* = 13)	CS IV (*n* = 7)
		Median: 0.793 IQR: 0.246–3.203	Median: 0.292 IQR: 0.0232–0.694	Median: 0.421 IQR: 0.023–1.086	Median: 0.329 IQR: 0.151–0.793
		Mann–Whitney U test *p* > 0.05		Mann–Whitney U test *p* > 0.05	
Plasma hsa-miR-22-3p (*n* = 16)	Median: 0.258 IQR: 0.111–2.025	Male (*n* = 8)	Female (*n* = 8)	CS III (*n* = 8)	CS IV (*n* = 8)
		Median: 0.255 IQR: 0.109–2.1	Median: 0.362 IQR: 0.111–2.025	Median: 0.392 IQR: 0.193–2.476	Median: 0.190 IQR: 0.063–1.65
		Mann–Whitney U test *p* > 0.05		Mann–Whitney U test *p* > 0.05	

There were no statistically significant differences between expression levels based on sample source (Mann–Whitney U test *p* > 0.05). The expression of has-miR-885-5p was not statistically significantly correlated with BMI and age, regardless of sample source (Spearman’s rho with *p* > 0.05).

**Table 5:** Analysis of selected prognostic parameters with the Cox proportional hazards model.

Variables	Beta Coefficient	Standard Error	*p*-Value	Hazard Ratio	Confidence Interval (95%)
Age	0.0674	0.0266	0.011	1.0696	1.0158–1.1264
Clinical stage	0.2393	0.4003	*p* > 0.05	1.0228	0.4295–2.4363
CA19-9	0.0001	0.0001	0.024	1.0002	1.0001–1.0003
CRP	0.0087	0.0033	0.008	1.0087	1.0022–1.0153
NLR	0.1382	0.053	0.022	1.1482	1.0354–1.2734
Overall model fit	Chi-square: 227,996, *p* = 0.0004				

## Data Availability

The data used to support the findings of this study are available from the corresponding author upon request.

## Supplementary Materials

The following supporting information can be downloaded at: https://www.mdpi.com/article/10.3390/cimb47010027/s1.
