# Supplementary material for: Serum hsa-miR-22-3p, hsa-miR-885-5p, Lipase-to-Amylase Ratio, C-Reactive Protein, CA19-9, and Neutrophil-to-Lymphocyte Ratio as Prognostic Factors in Advanced Pancreatic Ductal Adenocarcinoma
Source: Curr Issues Mol Biol. 2025 Jan 3;47(1):27. doi: 10.3390/cimb47010027 (PMC11763715; doi:10.3390/cimb47010027)

Supplementary Table S1. Mixture composition and conditions of reactions.

	Component	Volume [μl]	Reaction conditions			
			**Step**	**Temperature [°C]**	**Time [sec]**	**Repeats**
**Poly(A) tailing reation**	10x Poly(A) Buffer	0.5				
	ATP	0.5	Polyadenylation	37	2700	1
	Poly(A) Enzyme	0.3	Stop reaction	65	600	1
	RNase-free water	1.7	Hold	4	∞	
	RNA	2				
	Total volume	5				
**Adaptor ligation reaction**	5x DNA Ligase Buffer	3				
	50% PEG 8000	4.5				
	25x Ligation Adaptor	0.6	Ligation	16	360	1
	RNA Ligase	1.5	Hold	4	∞	
	RNase-free water	0.4				
	Poly(A) reaction product	5				
	Total volume	15				
**Reverse transcription reaction**	5x RT Buffer	6				
	dNTP Mix (25nM each)	1.2				
	20x Universal RT primer	1.5	Reverse transcription	42	900	1
	10x RT Enzyme Mix	3	Stop reaction	85	300	1
	RNase-free water	3.3	Hold	4	∞	
	Adaptor ligation product	15				
	Total volume	30				
**miR-Amp reaction**	2x miR-Amp Master Mix	25	Enzyme activation	95	300	1
	20x miR-Amp Primer Mix	2.5	Denature	95	3	14
	RNase-free water	17.5	Anneal/Extend	60	30	
	Reverse transcription product	5	Stop reaction	99	600	1
	Total volume	50	Hold	4	∞	

Supplementary Table S2. Influence of sex, performance status, T-status and N-status, history of pancreatic and biliary tract treatments, medical history, medication use, tobacco and alcohol use on potential prognostic factors.

Variables	CA19-9 median values [U/ml]	CRP median values [mg/dL]	LAR median values	NLR median values	PLR median values	LMR median values	serum hsa-miR 22-3p median values	plasma hsa-miR 22-3p median values	serum hsa-miR 885-5p median values	plasma hsa-miR 885-5p median values
**Sex**	Male (n=22): 760.5<br>Female (n=28): 815.7<br>Mann–Whitney U test p>0.05	Male (n=20): 10.5<br>Female (n=28): 4.9<br>Mann–Whitney U test p>0.05	Male (n=22): 0.72<br>Female (n=27): 0.63<br>Mann–Whitney U test p>0.05	Male (n=22):3.34<br>Female (n=28): 2.68<br>Mann–Whitney U test p>0.05	Male (n=22): 161.54<br>Female (n=28): 125.07<br>Mann–Whitney U test p>0.05	Male (n=22): 2.32<br>Female (n=28): 3.47<br>Mann–Whitney U test p>0.05	Male (n=8): 0.793<br>Female (n=13): 0.291<br>Mann–Whitney U test p>0.05	Male (n=8): 0.254<br>Female (n=8): 0.362<br>Mann–Whitney U test p>0.05	Male (n=14): 0.013<br>Female (n=15): 0.051<br>Mann–Whitney U test p>0.05	Male (n=12): 0.037<br>Female (n=15): 0.119<br>Mann–Whitney U test p>0.05
**Performance status - ECOG**	0 (n=11): 1128.00<br>1 (n=26): 75.56<br>2 (n=10): 1374.5<br>3 (n=1): 10000	0 (n=11): 2.9<br>1 (n=24): 5.45<br>2 (n=10): 22.35<br>3 (n=3): 67.0<br>**Kruskal-Wallis ANOVA p=0.031**	0 (n=10): 0.77<br>1 (n=26): 0.63<br>2 (n=10): 0.59<br>3 (n=3): 0.70<br>Kruskal-Wallis ANOVA p>0.05	0 (n=11): 2.47<br>1 (n=26): 2.82<br>2 (n=10): 3.59<br>3 (n=3): 3.68<br>Kruskal-Wallis ANOVA p>0.05	0 (n=11): 113.49<br>1 (n=26): 132.66<br>2 (n=10): 188.71<br>3 (n=3): 96.08<br>Kruskal-Wallis ANOVA p>0.05	0 (n=11): 2.95<br>1 (n=26): 2.78<br>2 (n=10): 2.90<br>3 (n=3): 2.94<br>Kruskal-Wallis ANOVA p>0.05	0 (n=3): 0.329<br>1 (n=12): 0.625<br>2 (n=3): 0.246<br>3 (n=2): 2.83<br>Kruskal-Wallis ANOVA p>0.05	0 (n=2): 0.034<br>1 (n=10): 0.255<br>2 (n=1): 0.497<br>3 (n=3): 1.29	0(n=9): 0.019<br>1 (n=14): 0.013<br>2 (n=5): 0.062<br>3 (n=1): 0.001	0 (n=8): 0.024<br>1 (n=13): 0.11<br>2 (n=4): 0.19<br>3 (n=2): 0.041
**TNM – T status**	T2 (n=6): 1369.91<br>T3 (n=10): 978<br>T4 (n=34): 627<br>Kruskal-Wallis ANOVA p>0.05	T2 (n=6): 63.9<br>T3 (n=9): 5.7<br>T4 (n=33): 4.4<br>**Kruskal-Wallis ANOVA p=0.02**	T2 (n=6): 0.73<br>T3 (n=9): 0.7<br>T4(n=34): 0.63<br>Kruskal-Wallis ANOVA p>0.05	T2 (n=6): 3.31<br>T3 (n=10): 3.10<br>T4(n=34): 2.61<br>Kruskal-Wallis ANOVA p>0.05	T2 (n=6): 131.83<br>T3 (n=10): 133.5<br>T4(n=34): 132.66<br>Kruskal-Wallis ANOVA p>0.05	T2 (n=6): 2.74<br>T3 (n=10): 2.39<br>T4(n=34): 3.15<br>Kruskal-Wallis ANOVA p>0.05	T2 (n=4): 1.622<br>T3 (n=3): 0.33<br>T4(n=13): 0.292<br>Kruskal-Wallis ANOVA p>0.05	T2 (n=5): 1.29<br>T3 (n=4): 1.565<br>T4(n=7): 0.158<br>Kruskal-Wallis ANOVA p>0.05	T2 (n=1): 0.001<br>T3 (n=7): 0.821<br>T4(n=21): 0.03	T2 (n=3): 0.082<br>T3 (n=7): 0.019<br>T4(n=17): 0.106<br>Kruskal-Wallis ANOVA p>0.05
**TNM –**	N0 (n=19):	N0 (n=18):	N0 (n=19):	N0 (n=19):	N0 (n=19):	N0 (n=19):	N0 (n=8):	N0 (n=6):	N0 (n=10):	N0 (n=12):

**Nstatus**	761<br>N1 (n=21): 494.6<br>N2 (n=6): 1889<br>N3 (n=3): 3064.1<br>Kruskal-Wallis ANOVA p>0.05	5.7<br>N1 (n=20): 4.9<br>N2 (n=6): 6.55<br>N3 (n=2): 162.6	0.70<br>N1 (n=20): 0.80<br>N2 (n=6): 0.33<br>N3 (n=2): 0.56	2.93<br>N1 (n=21): 2.55<br>N2 (n=6): 2.43<br>N3 (n=2): 4.52	110.62<br>N1 (n=21): 156.25<br>N2 (n=6): 111.57<br>N3 (n=2): 244.92	2.66<br>N1 (n=21): 3.19<br>N2 (n=6): 3.61<br>N3 (n=2): 1.31	0.784<br>N1 (n=7): 0.023<br>N2 (n=3): 0.23<br>N3 (n=2): 0.472	0.392<br>N1 (n=5): 0.158<br>N2 (n=3): 0.228<br>N3 (n=1): 0.158<br>Kruskal-Wallis ANOVA p>0.05	0.023<br>N1 (n=14): 0.013<br>N2 (n=3): 0.735<br>N3 (n=1): 0.078	0.131<br>N1 (n=13): 0.019<br>N2 (n=0):<br>N3 (n=1): 13.078
**History of cholecystectomy**	Yes (n=9): 928.9<br>No (n=41): 760<br>Mann–Whitney U test p>0.05	Yes (n=9): 6.2<br>No (n=39): 5.2<br>Mann–Whitney U test p>0.05	Yes (n=9): 0.762<br>No (n=40): 0.670<br>Mann–Whitney U test p>0.05	Yes (n=9): 2.42<br>No (n=41): 2.72<br>Mann–Whitney U test p>0.05	Yes (n=9): 135.47<br>No (n=41): 128.94<br>Mann–Whitney U test p>0.05	Yes (n=9): 3.27<br>No (n=41): 2.87<br>Mann–Whitney U test p>0.05	Yes (n=3): 0.694<br>No (n=17): 0.330<br>Mann–Whitney U test p>0.05	Yes (n=2): 0.248<br>No (n=14): 0.257	Yes (n=4): 0.267<br>No (n=25): 0.03<br>Mann–Whitney U test p>0.05	Yes (n=5): 0.142<br>No (n=22): 0.037<br>Mann–Whitney U test p>0.05
**History of palliative pancreatic bypass surgery**	Yes (n=4): 1575.8<br>No (n=46): 782.2<br>Mann–Whitney U test p>0.05	Yes (n=4): 12.1<br>No (n=44): 5.7<br>Mann–Whitney U test p>0.05	Yes (n=4): 0.66<br>No (n=45): 0.7<br>Mann–Whitney U test p>0.05	Yes (n=4): 3.11<br>No (n=46): 2.70<br>Mann–Whitney U test p>0.05	Yes (n=4): 174.28<br>No (n=46): 129.39<br>Mann–Whitney U test p>0.05	Yes (n=4): 3.41<br>No (n=46): 2.90<br>Mann–Whitney U test p>0.05	Yes (n=0)<br>No (n=20): 0.38	Yes (n=0)<br>No (n=16): 0.26	Yes (n=2): 0.77<br>No (n=27): 0.019	Yes (n=3): 0.21<br>No (n=24): 0.037
**History of billiary stent placement**	Yes (n=24): 1028.5<br>No (n=28): 481.3<br>Mann–Whitney U test p>0.05	Yes (n=24): 5.7<br>No (n=24): 7.2<br>Mann–Whitney U test p>0.05	Yes (n=23): 0.59<br>No (n=26): 0.77<br>Mann–Whitney U test p>0.05	Yes (n=24): 2.63<br>No (n=26): 2.94<br>Mann–Whitney U test p>0.05	Yes (n=24): 132.7<br>No (n=26): 135.9<br>Mann–Whitney U test p>0.05	Yes (n=24): 3.23<br>No (n=26): 2.07<br>Mann–Whitney U test p>0.05	Yes (n=12): 0.493<br>No (n=8): 0.375<br>Mann–Whitney U test p>0.05	Yes (n=12): 0.193<br>No (n=8): 1.148<br>Mann–Whitney U test p>0.05	Yes (n=14): 0.032<br>No (n=15): 0.03<br>Mann–Whitney U test p>0.05	Yes (n=12): 0.011<br>No (n=15): 0.172<br>Mann–Whitney U test p>0.05
**History of pulmonary disease**	Yes (n=4): 233.5	**Yes (n=4): 37.8**	Yes (n=4): 0.51	Yes (n=4): 4.19	Yes (n=4): 138.5	Yes (n=4): 1.63	Yes (n=2): 0.28	Yes (n=3): 0.222	Yes (n=0): 0.222	Yes (n=1): 0.0002

	No (n=46): 815.7<br>Mann–Whitney U test p>0.05	**No (n=44): 5.5**<br>**Mann–Whitney U test p=0.03**	No (n=45): 0.7<br>Mann–Whitney U test p>0.05	No (n=46): 2.71<br>Mann–Whitney U test p>0.05	No (n=46): 132.7<br>Mann–Whitney U test p>0.05	No (n=46): 3.02<br>Mann–Whitney U test p>0.05	No (n=18): 0.375	No (n=13): 0.287<br>Mann–Whitney U test p>0.05	No (n=29): 0.03	No (n=26): 0.094
**History of diabetes**	Yes (n=19): 468<br>No (n=31): 803.4<br>Mann–Whitney U test p>0.05	Yes (n=19): 14.6<br>No (n=29): 5.9<br>Mann–Whitney U test p>0.05	**Yes (n=19): 0.98**<br>**No (n=30): 0.60**<br>**Mann–Whitney U test p=0.03**	Yes (n=19): 2.94<br>No (n=31): 2.71<br>Mann–Whitney U test p>0.05	Yes (n=19): 116.61<br>No (n=31): 150.87<br>Mann–Whitney U test p>0.05	Yes (n=19): 3.08<br>No (n=31): 2.87<br>Mann–Whitney U test p>0.05	Yes (n=8): 0.625<br>No (n=12): 0.311<br>Mann–Whitney U test p>0.05	Yes (n=7): 1.290<br>No (n=9): 0.157<br>Mann–Whitney U test p>0.05	Yes (n=11): 0.012<br>No (n=18): 0.034<br>Mann–Whitney U test p>0.05	Yes (n=7): 0.082<br>No (n=20): 0.083<br>Mann–Whitney U test p>0.05
**History of cardiovascular disease**	Yes (n=37): 928<br>No (n=13): 118<br>Mann–Whitney U test p>0.05	Yes (n=36): 5.7<br>No (n=12): 14.2<br>Mann–Whitney U test p>0.05	Yes (n=36): 0.67<br>No (n=13): 0.69<br>Mann–Whitney U test p>0.05	Yes (n=37): 2.72<br>No (n=13): 2.70<br>Mann–Whitney U test p>0.05	Yes (n=37): 135.47<br>No (n=13): 129.85<br>Mann–Whitney U test p>0.05	Yes (n=37): 2.95<br>No (n=13): 2.53<br>Mann–Whitney U test p>0.05	Yes (n=14): 0.443<br>No (n=6): 0.286<br>Mann–Whitney U test p>0.05	Yes (n=13): 0.228<br>No (n=3): 2.04<br>Mann–Whitney U test p>0.05	Yes (n=19): 0.03<br>No (n=10): 0.047<br>Mann–Whitney U test p>0.05	Yes (n=19): 0.019<br>No (n=8): 0.261<br>Mann–Whitney U test p>0.05
**History of anticoagulant medications intake**	Yes (n=10): 127.5<br>No (n=40): 781.7<br>Mann–Whitney U test p>0.05	Yes (n=9): 27.8<br>No (n=39): 5.6<br>Mann–Whitney U test p>0.05	Yes (n=10): 0.75<br>No (n=39): 0.65<br>Mann–Whitney U test p>0.05	Yes (n=10): 3.93<br>No (n=40): 2.70<br>Mann–Whitney U test p>0.05	Yes (n=10): 138.16<br>No (n=40): 132.66<br>Mann–Whitney U test p>0.05	Yes (n=10): 2.56<br>No (n=40): 3.02<br>Mann–Whitney U test p>0.05	**Yes (n=4): 2.717**<br>**No (n=16): 0.269**<br>**Mann–Whitney U test p=0.01**	Yes (n=4): 0.255<br>No (n=12): 0.362<br>Mann–Whitney U test p>0.05	Yes (n=4): 0.002<br>No (n=25): 0.038<br>Mann–Whitney U test p>0.05	Yes (n=5): 0.082<br>No (n=22): 0.076<br>Mann–Whitney U test p>0.05
**History of hypotensive medications**	Yes (n=32): 878<br>No (n=18): 481.3<br>Mann–Whitney U test p>0.05	Yes (n=31): 5.7<br>No (n=17): 5.6<br>Mann–Whitney U test p>0.05	Yes (n=11): 0.65<br>No (n=18): 0.73<br>Mann–Whitney U test p>0.05	Yes (n=32): 2.84<br>No (n=18): 2.57<br>Mann–Whitney U test p>0.05	Yes (n=32): 2.84<br>No (n=18): 2.57<br>Mann–Whitney U test p>0.05	Yes (n=32): 2.92<br>No (n=18): 3.15<br>Mann–Whitney U test p>0.05	Yes (n=13): 0.556<br>No (n=7): 0.246<br>Mann–Whitney U test p>0.05	Yes (n=12): 0.258<br>No (n=4): 1.1<br>Mann–Whitney U test p>0.05	Yes (n=16): 0.034<br>No (n=13): 0.015<br>Mann–Whitney U test p>0.05	Yes (n=17): 0.046<br>No (n=10): 0.212<br>Mann–Whitney U test p>0.05
**History of oral hypoglycemic**	Yes (n=13):	Yes (n=13): 14.6	Yes (n=13):	Yes (n=13): 3.21	Yes (n=13):	Yes (n=13):	Yes (n=4): 1.393	Yes (n=5): 0.228	Yes (n=7): 0.042	Yes (n=4): 0.213

**medications**	118<br>No (n=37): 828<br>Mann–Whitney U test p>0.05	No (n=35): 5.4<br>Mann–Whitney U test p>0.05	0.81<br>No (n=36): 0.64<br>Mann–Whitney U test p>0.05	No (n=37): 2.71<br>Mann–Whitney U test p>0.05	155.68<br>No (n=37): 129.85<br>Mann–Whitney U test p>0.05	2.47<br>No (n=37): 2.95<br>Mann–Whitney U test p>0.05	No (n=16): 0.311<br>Mann–Whitney U test p>0.05	No (n=11): 0.287<br>Mann–Whitney U test p>0.05	No (n=22): 0.025<br>Mann–Whitney U test p>0.05	No (n=23): 0.052<br>Mann–Whitney U test p>0.05
**History of alcohol usage**	Yes (n=9): 1128<br>No (n=41): 760<br>Mann–Whitney U test p>0.05	Yes (n=9): 5.4<br>No (n=39): 6.2<br>Mann–Whitney U test p>0.05	Yes (n=9): 0.70<br>No (n=40): 0.66<br>Mann–Whitney U test p>0.05	Yes (n=9): 2.94<br>No (n=41): 2.71<br>Mann–Whitney U test p>0.05	Yes (n=9): 150.87<br>No (n=41): 129.85<br>Mann–Whitney U test p>0.05	Yes (n=9): 3.42<br>No (n=41): 2.87<br>Mann–Whitney U test p>0.05	**Yes (n=3): 0.001**<br>**No (n=17): 0.556**<br>**Mann–Whitney U test p=0.01**	Yes (n=2): 2.48<br>No (n=41): 0.225	Yes (n=6): 0.386<br>No (n=23): 0.019<br>Mann–Whitney U test p>0.05	Yes (n=6): 0.083<br>No (n=21): 0.082<br>Mann–Whitney U test p>0.05
**History of smoking**	Yes (n=21): 761<br>No (n=29): 828<br>Mann–Whitney U test p>0.05	Yes (n=21): 5.6<br>No (n=27): 5.7<br>Mann–Whitney U test p>0.05	Yes (n=21): 0.81<br>No (n=28): 0.64<br>Mann–Whitney U test p>0.05	Yes (n=21): 2.7<br>No (n=29): 2.76<br>Mann–Whitney U test p>0.05	Yes (n=21): 158.94<br>No (n=29): 150.87<br>Mann–Whitney U test p>0.05	Yes (n=21): 2,87<br>No (n=29): 2.95<br>Mann–Whitney U test p>0.05	Yes (n=10): 0.512<br>No (n=10): 0.356<br>Mann–Whitney U test p>0.05	Yes (n=9): 0.158<br>No (n=7): 0.497<br>Mann–Whitney U test p>0.05	Yes (n=10): 0.027<br>No (n=19): 0.03<br>Mann–Whitney U test p>0.05	Yes (n=8): 0.067<br>No (n=19): 0.082<br>Mann–Whitney U test p>0.05

Supplementay Table S3. Proportional hazard assumption testes results.

Variables	chi-square	P value
**Age**	**7.318**	**0.007**
**Clinical stage**	0.772	>0.05
**CA19-9**	0.656	>0.05
**CRP**	1.733	>0.05
**NLR**	1.467	>0.05
**General test result**	9.609	>0.05

Firgure S1. Scaled Schoenfeld residuals for age, time in days

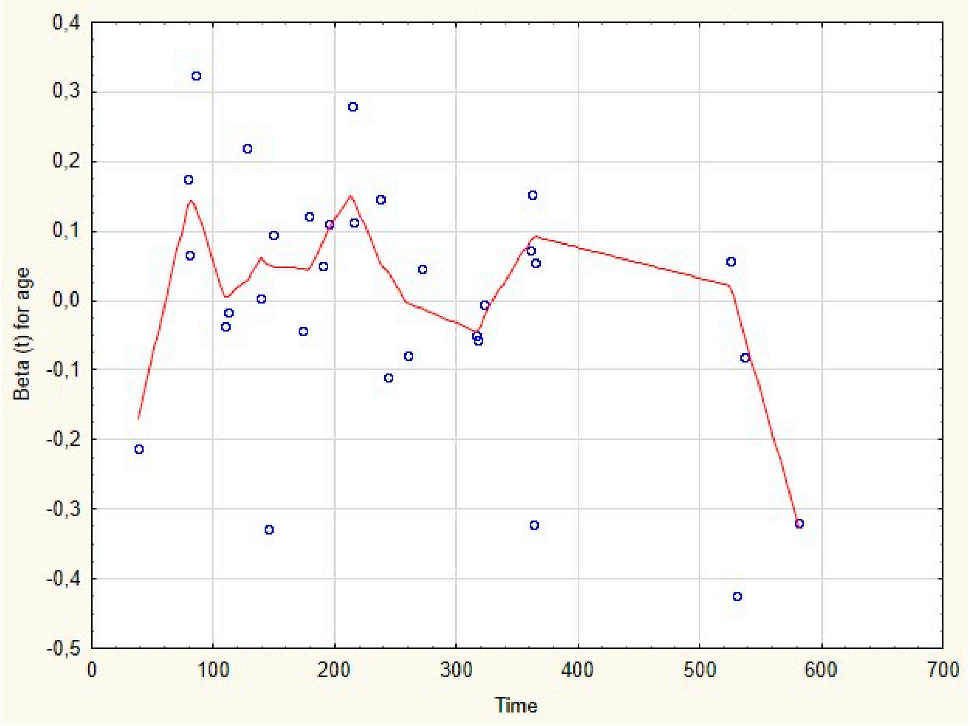

Firgure S2. Scaled Schoenfeld residuals for clinical stage, time in days

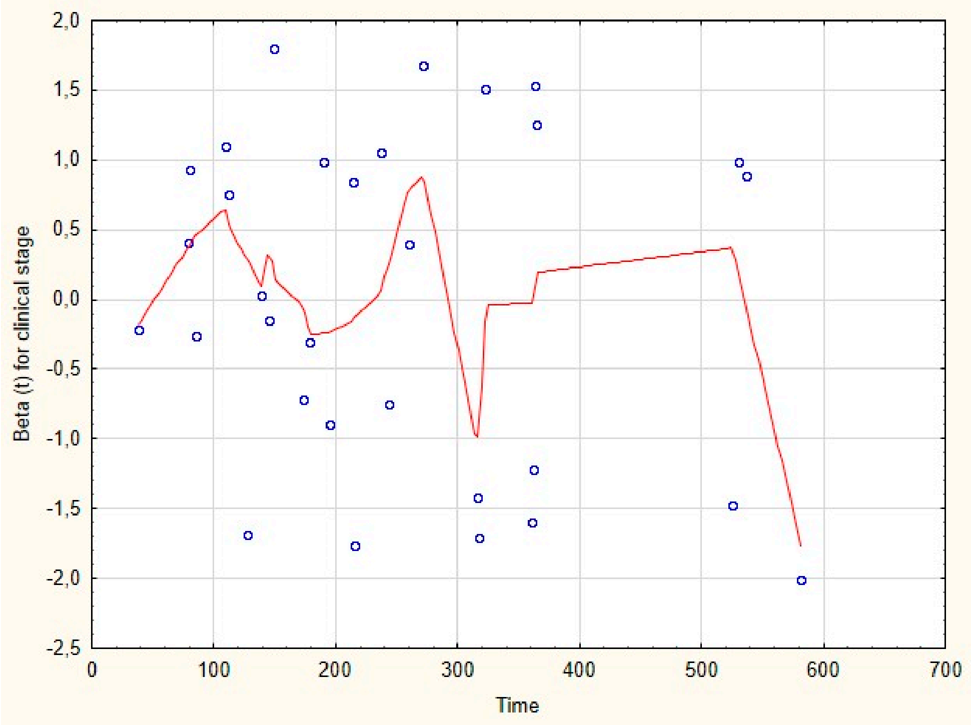

Firgure S3. Scaled Schoenfeld residuals for CA 19-9, time in days

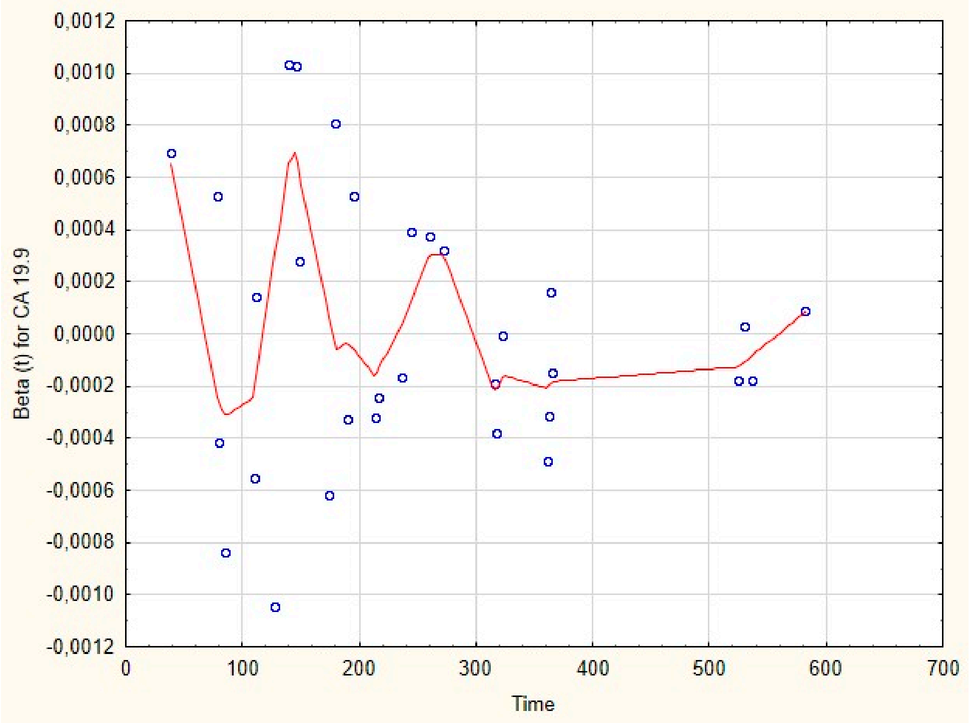

Firgure S4. Scaled Schoenfeld residuals for CRP, time in days

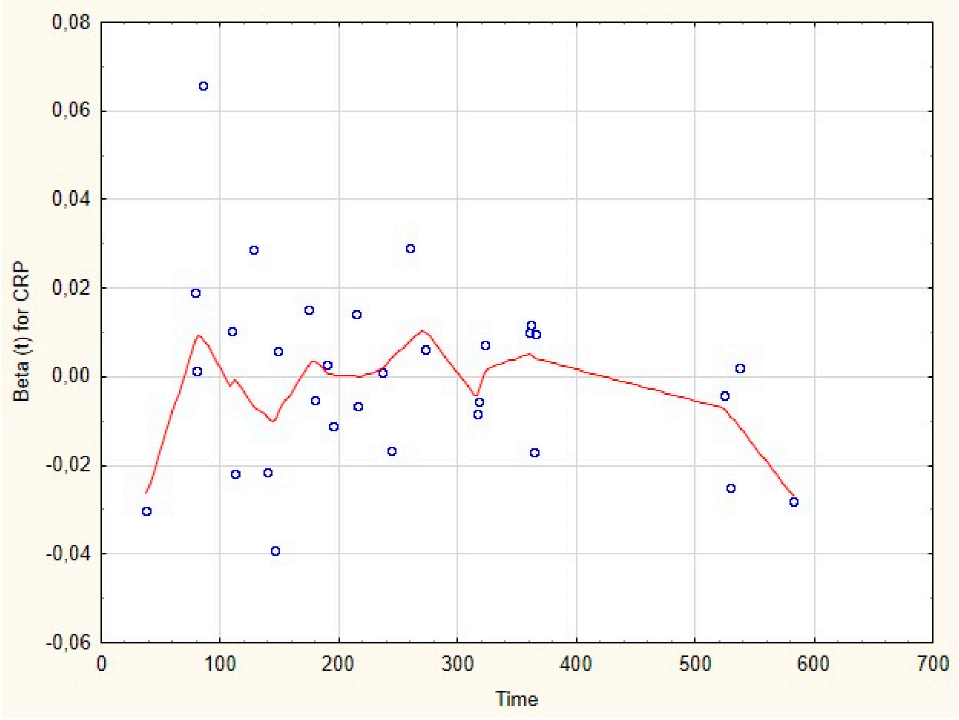

Firgure S5. Scaled Schoenfeld residuals for NLR, time in days

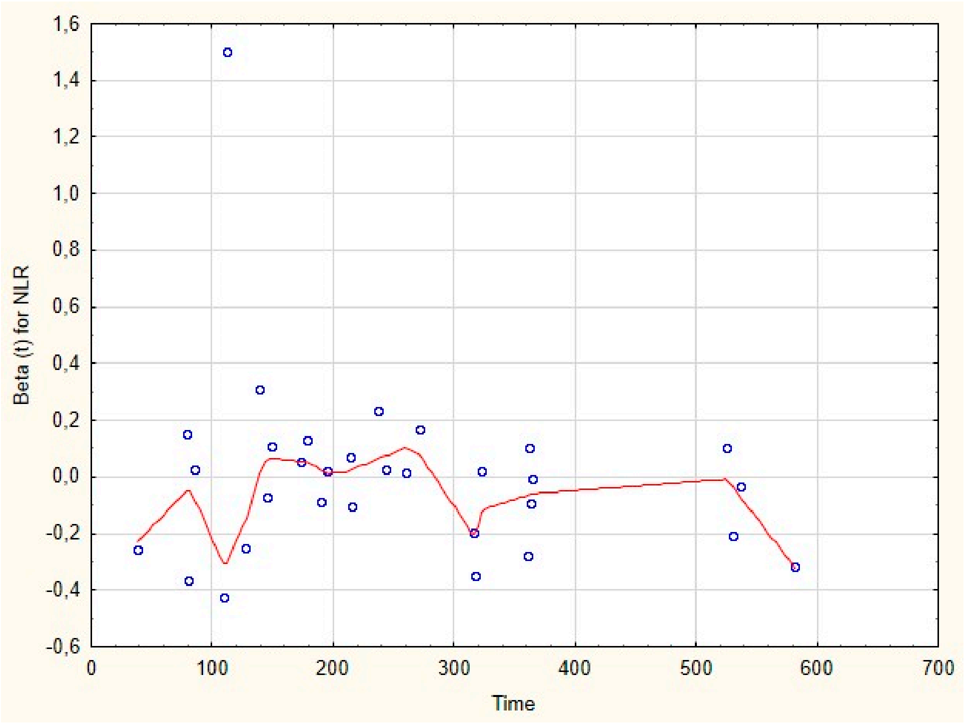

Supplement: Supplementary file 1 [file cimb-47-00027-s001.zip › cimb-3383892-supplementary.pdf]
